# Karyomegalic interstitial nephritis with a novel FAN1 gene mutation and concurrent ALECT2 amyloidosis

**DOI:** 10.1186/s12882-020-01733-9

**Published:** 2020-02-28

**Authors:** Steven Law, Julian Gillmore, Janet A. Gilbertson, Paul Bass, Alan D. Salama

**Affiliations:** 1 UCL Department of Renal Medicine, Centre for Experimental Nephrology, Royal Free Hospital, NW3 2PF, London, UK; 2 National Amyloidosis Centre, Division of Medicine, University College London, NW3 2PF, London, UK; 3 Department of Pathology, Royal Free Hospital, NW3 2PF, London, UK

**Keywords:** Karyomegalic interstitial nephritis, Interstitial nephritis, Leukocyte chemotactic factor 2 amyloidosis, ALECT2, Amyloidosis, Chronic kidney disease

## Abstract

**Background:**

Karyomegalic interstitial nephritis (KIN) is a rare hereditary cause of chronic kidney disease. It typically causes progressive renal impairment with haemoproteinuria requiring renal replacement therapy before 50 years of age. It has been associated with mutations in the Fanconi anaemia-associated nuclease 1 (FAN1) gene and has an autosomal recessive pattern of inheritance. Leukocyte chemotactic factor 2 amyloidosis (ALECT2) is the third most common cause of amyloid nephropathy presenting with chronic kidney disease and variable proteinuria. We report a novel mutation in the FAN1 gene causing KIN and to our knowledge, the first case of concurrent KIN and ALECT.

**Case presentation:**

We describe the case of 44 year old Pakistani woman, presenting with stage four non-proteinuric chronic kidney disease, and a brother on dialysis. Renal biopsy demonstrated KIN and concurrent ALECT2. Genetic sequencing identified a novel FAN1 mutation as the cause of her KIN and she is being managed conservatively for chronic kidney disease. Her brother also had KIN with no evidence of amyloidosis and is being worked up for kidney transplantation.

**Conclusion:**

This case highlights two rare causes of chronic kidney disease considered underdiagnosed in the wider population due to their lack of proteinuria, and may contribute to the cohort of patients reaching end stage renal disease without a renal biopsy. We report a novel mutation of the FAN1 gene causing KIN, and report the first case of concurrent KIN and ALECT2. This case highlights the importance of renal biopsy in chronic kidney disease of unclear aetiology which has resulted in a diagnosis with implications for kidney transplantation and family planning.

## Summary

We present the case of a patient with chronic kidney disease due to a novel Fanconi anaemia-associated nuclease 1 (FAN1) mutation causing karyomegalic interstitial nephritis (KIN), and concurrent leukocyte chemotactic factor 2 amyloidosis (ALECT2). This is the first reported case of a patient homozygous/bi-allelic for FAN1 *c.1899deletion* causing KIN and the first time these two rare causes of chronic kidney disease have been described in the same patient, who unusually had no proteinuria. Both diagnoses generally present with progressive chronic kidney disease and variable proteinuria requiring a biopsy for diagnosis and may be underdiagnosed in the wider population.

## Case presentation

A 44 year old woman from Pakistan presented to our service with chronic kidney disease of unknown aetiology. She was asymptomatic and had been told by her local hospital that she had chronic kidney disease five years earlier. She had gestational diabetes during pregnancy with her son and had recently been diagnosed with type two diabetes, with no complications, and started on repaglinide. She had hypothyroidism and was on thyroxine replacement therapy for 10 years but no other previous medical history. Her other medications consisted of sodium bicarbonate 600 mg twice daily, repaglinide 0.5 mg twice daily and allopurinol 150 mg daily. She took no over the counter or herbal medicines.

The patient’s 52 year old brother was found to have biopsy proven karyomegalic interstitial nephritis and had progressed to end stage renal disease but had not been genotyped; his biopsy had no features of amyloidosis. In addition another older brother and sister were noted to have normal kidney function as did her son. Her parents were first cousins and her mother had diabetes and hypertension; her father died from prostate carcinoma aged 73.

Clinical examination of all systems was unremarkable with a blood pressure of 120/80 mmHg and heart rate of 72 beats per minute.

Urinalysis showed no blood or protein, microscopy revealed no leucocytes, urine protein creatinine ratio was 17 mg/mmol (NR 0–30) and no bence jones protein was detected. Haemoglobin was 110 g/L with normal white blood cells, and platelets. Creatinine was 223 μmol/l, a MDRD estimated glomerular filtration rate (eGFR) of 22 ml/min/1.73^2^ with a normal sodium, potassium, calcium, phosphate, bilirubin and alkaline phosphatase; parathyroid hormone was elevated at 8.4 pmol/L. Creatinine five years earlier was 121 μmol/l with an eGFR of 45 ml/min/1.73m^2^. Immunological screen was negative, in particular autoantibodies (ANA, ANCA, anti-dsDNA, ENA)were negative, complement C3 and C4 were in the normal range and immunoglobulins showed a mildly elevated IgG at 16.5 g/L (NR 7–16), but normal IgA and IgM levels and protein electrophoresis showed a polyclonal increase in gammaglobulins. Serum free light chains were kappa 33.4 mg/L and lamda 19.7 mg/l, with a ratio of 1.7 in keeping with renal impairment.

Ultrasound demonstrated 8.5 cm unobstructed kidneys with increased echogenicity of the parenchyma.

Sequence analysis using the Blueprint Genetics FAN1 single gene test identified a homozygous single base pair deletion c.1899del, p.(Cys633Trpfs*9) in the FAN1 gene (transcript number: NM_014967.4). This was confirmed with bi-directional Sanger sequencing and performed in a CLIA-certified laboratory accredited by the College of American Pathologists.

**Fig. 1:**
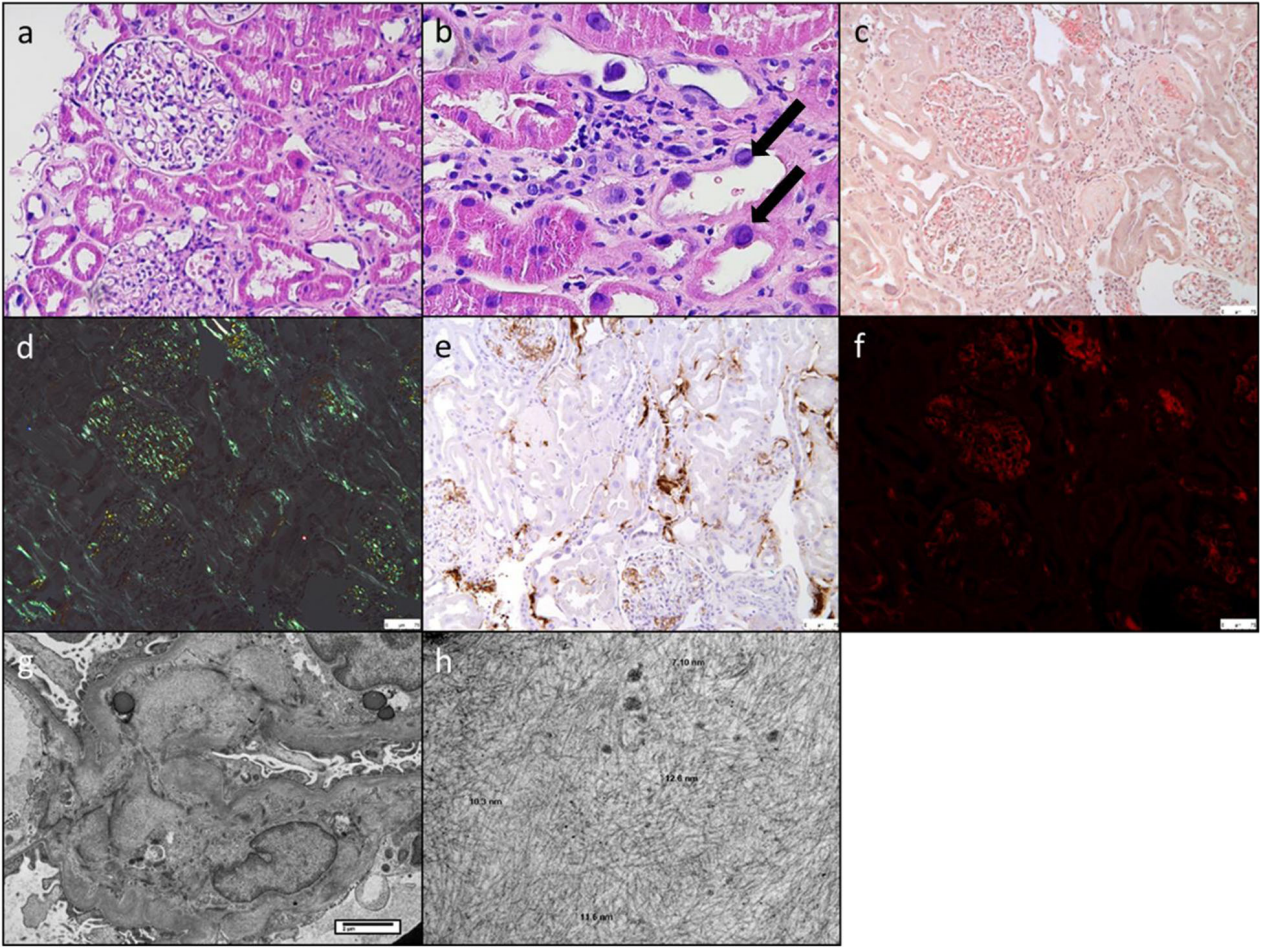
**a** and **b**) H&E staining with marked irregularity of the tubules, tubular cell nuclear pleomorphism and prominent nucleoli (black arrows). **c**) Mesangial and interstitial congo-red positivity. **d**) Classic apple green birefringence under polarised light. **e**) Positive immunohistochemistry after staining with anti-LECT2 antibody. **f**) Congo-red staining under fluorescent light. **g** and **h**) Randomly orientated and non-branching fibrils of 7–11 nm in the glomerular basement membrane and mesangium (*Electron microscopy images courtesy of Leicester Royal infirmary Electron Microscopy department*)

Renal biopsy demonstrated 28 glomeruli of which seven were globally sclerosed, with some glomeruli displaying congophilic material in the mesangial areas. In the interstitium there was approximately 10% chronic interstitial fibrosis and surrounding areas of light interstitial inflammatory cell infiltrate. There was marked irregularity of the tubules, with tubular cell nuclear pleomorphism and prominent nucleoli (Fig. 1: b). In addition to the mesangium, there were small foci of interstitial amyloid on the congo-red stain with demonstrated apple green birefringence under polarised light (Fig. 1: c and d). There was no immunoglobulin or complement deposition, and staining for serum amyloid A, kappa and lambda light chains was negative. Immunostaining for LECT2 was positive (Fig. 1: e). On electron microscopy there were no electron dense deposits but there were randomly orientated and non-branching fibrils 7–11 nm in size found in the glomerular basement membranes and mesangial areas (Fig. 1: g, h).

**Fig. 2:**
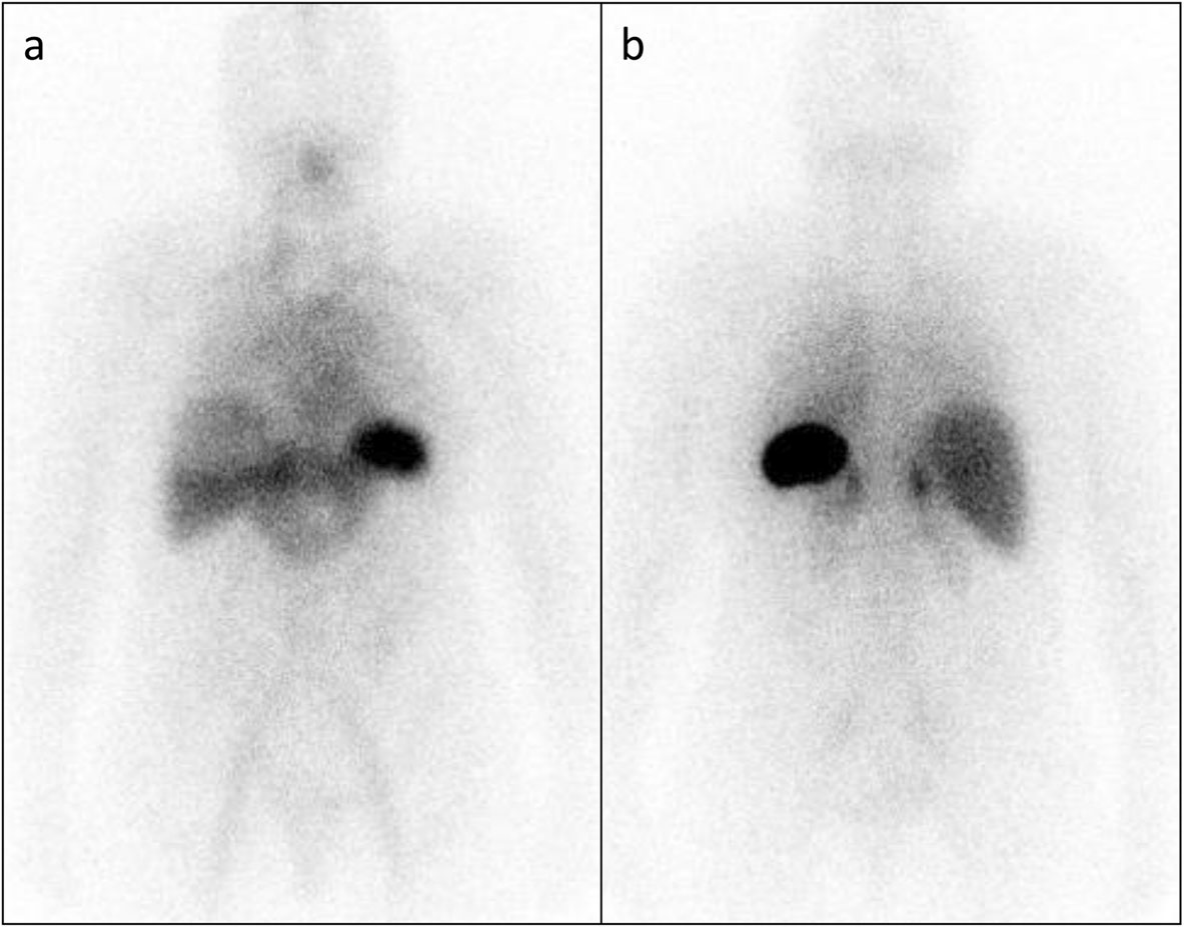
Whole body anterior and posterior ^123^I-labelled SAP scans showing a moderate amyloid load in the spleen, adrenal glands and kidneys with equivocal liver uptake

Serum amyloid P scintigraphy showed a moderate amyloid load in the spleen and adrenal glands with equivocal renal uptake (Fig. 2). Her echocardiogram demonstrated no features of cardiac amyloid.

A diagnosis of KIN secondary to a novel homozygous single base pair deletion *c.1899del* in the FAN1 gene with concurrent ALECT2 was made, and the patient is currently being managed supportively for her chronic kidney disease. Her brother is set to undergo live donor transplantation from his wife.

## Discussion and conclusions

KIN is a rare condition presenting with progressive renal dysfunction, haemoproteinuria and a family history of renal disease. There is no clear sex or ethnic predilection and patients often present in their 20’s with recurrent respiratory infections [1]. End stage renal failure predictably occurs before 50 years of age [2]. Karyomegalic cells are often limited to the kidney but may be present in the liver, lungs, skin, GI tract, myocardium and brain, although these rarely manifest clinically. Diagnosis is confirmed with renal histology which typically shows chronic tubulointerstitial nephritis with characteristic enlargement of tubular nuclei on electron microscopy with irregular outlines, course chromatin and no viral inclusions [3]. Recent evidence has shown an association between mutations in the FAN1 gene and KIN. The FAN1 gene encodes a DNA endo- and exonuclease protein which acts within the Fanconi anaemia DNA damage response pathway to repair DNA. The FAN1 gene is located on chromosome 15 and mutations are inherited in an autosomal recessive manner [4]. In this case the patient was homozygous for FAN1 *c.1899deletion* which produces a frameshift and a premature stop codon at position 9. There are no patients homozygous for this deletion in the Genome Aggregation Database; 8 patients are heterozygous (*n* > 120,000 exomes) [5]. There is no specific treatment for KIN at present but genetic counselling for affected families should be considered.

ALECT2 is the third most common cause of renal amyloidosis behind AL and AA amyloidosis [6]. The average age of presentation is 65 years with renal dysfunction and variable proteinuria including nephrotic syndrome in 10% [7, 8]. ALECT2 predominantly affects the kidneys but can involve the liver, spleen, prostate and pancreas [6]. Histology characteristically demonstrates marked congophilia of the glomerular, interstitial and vascular compartments [6]. Immunohistochemistry with anti-LECT2 antibody confirms the diagnosis, but weak staining can lead to false negatives; laser microdissection and mass spectrometry may increase sensitivity [9]. There is no clear genetic cause although recent case series show a higher prevalence in Hispanic, Punjabi, Egyptian, Sudanese and Chinese populations [8]. A series of 24 patients demonstrated a median eGFR of 33 ml/min/1.73m^2^, proteinuria of 0.5 g per 24 h and mean age of 62 years. Mean eGFR loss was 4.2 ml/min/1.73m^2^ per year without significant progression of proteinuria. The median time from diagnosis to end stage renal disease was 8.2 years [10]. Currently there are no targeted treatments and management is supportive. A series of five patients who underwent renal transplantation highlighted good early graft outcomes with recurrence in one patient at six months [11]. Due to the slowly progressive nature and minimal proteinuria in ALECT2 the condition is felt to be underdiagnosed especially in high risk populations.

In summary, we present the first reported case of concurrent LECT2 amyloidosis and KIN combined with a novel deletion in the FAN1 gene. Interestingly, the patient and her brother who both had KIN, were disparate for ALECT2. From the patient’s perspective, there is now a clear diagnosis and a means to screen other family members. In addition, the dual diagnoses complicates future transplant outcome in which there is now a possibility of recurrent ALECT2.

## Abbreviations

ALECT2: Leukocyte chemotactic factor 2 amyloidosis
eGFR: Estimated glomerular filtration rate
FAN1: Fanconi anaemia-associated nuclease 1
KIN: Karyomegalic interstitial nephritis
MDRD: Modification of Diet in Renal Disease
